# The effect of surgical trauma on circulating free DNA levels in cancer patients—implications for studies of circulating tumor DNA

**DOI:** 10.1002/1878-0261.12729

**Published:** 2020-06-16

**Authors:** Tenna V. Henriksen, Thomas Reinert, Emil Christensen, Himanshu Sethi, Karin Birkenkamp‐Demtröder, Mikail Gögenur, Ismail Gögenur, Bernhard G. Zimmermann, The IMPROVE Study Group, Lars Dyrskjøt, Claus L. Andersen

**Affiliations:** ^1^ Department of Molecular Medicine Aarhus University Hospital Aarhus N Denmark; ^2^ Natera Inc. San Carlos CA USA; ^3^ Center for Surgical Sciences Zealand University Hospital Køge Denmark

**Keywords:** bladder cancer, cell‐free DNA, circulating tumor DNA, colorectal cancer, trauma

## Abstract

Detection of circulating tumor DNA (ctDNA) post‐treatment is an emerging marker of residual disease. ctDNA constitutes only a minor fraction of the cell‐free DNA (cfDNA) circulating in cancer patients, complicating ctDNA detection. This is exacerbated by trauma‐induced cfDNA. To guide optimal blood sample timing, we investigated the duration and magnitude of surgical trauma‐induced cfDNA in patients with colorectal or bladder cancer. DNA levels were quantified in paired plasma samples collected before and up to 6 weeks after surgery from 436 patients with colorectal cancer and 47 patients with muscle‐invasive bladder cancer. To assess whether trauma‐induced cfDNA fragments are longer than ordinary cfDNA fragments, the concentration of short (< 1 kb) and long (> 1 kb) fragments was determined for 91 patients. Previously reported ctDNA data from 91 patients with colorectal cancer and 47 patients with bladder cancer were used to assess how trauma‐induced DNA affects ctDNA detection. The total cfDNA level increased postoperatively—both in patients with colorectal cancer (mean threefold) and bladder cancer (mean eightfold). The DNA levels were significantly increased up to 4 weeks after surgery in both patient cohorts (*P* = 0.0005 and *P* ≤ 0.0001). The concentration of short, but not long, cfDNA fragments increased postoperatively. Of 25 patients with radiological relapse, eight were ctDNA‐positive and 17 were ctDNA‐negative in the period with trauma‐induced DNA. Analysis of longitudinal samples revealed that five of the negative patients became positive shortly after the release of trauma‐induced cfDNA had ceased. In conclusion, surgery was associated with elevated cfDNA levels, persisting up to 4 weeks, which may have masked ctDNA in relapse patients. Trauma‐induced cfDNA was of similar size to ordinary cfDNA. To mitigate the impact of trauma‐induced cfDNA on ctDNA detection, it is recommended that a second blood sample collected after week 4 is analyzed for patients initially ctDNA negative.

## Graphical abstract

We studied the change in circulating DNA levels resulting from cancer surgery. Surgical trauma caused an increase in the level of circulating DNA, which persisted up to 4 weeks after surgery. Detection of circulating tumor DNA after surgery was hampered by increased circulating DNA levels.

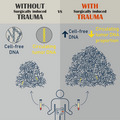

**Abbreviations**
cfDNA: cell‐free DNA
ctDNA: circulating tumor DNA
ddPCR: droplet digital PCR
VAF: variant allele frequency

## 1 Introduction

Analysis of circulating tumor DNA (ctDNA) in blood samples drawn after curatively intended cancer surgery is an emerging approach for identifying patients with molecular residual disease and very high risk for clinical disease recurrence (Chaudhuri *et al*., 2017; Christensen *et al*., 2019; Garcia‐Murillas *et al*., 2015; Reinert *et al*., 2016, 2019; Schøler et al., 2017). Trauma causes augmented cell death and has been reported to be associated with increased levels of cell‐free DNA (cfDNA) in the blood (Gögenur *et al*., 2017; Jackson Chornenki *et al*., 2019; Prakash *et al*., 2017; Qi *et al*., 2016; Ren *et al*., 2013). Trauma‐induced release of wild‐type DNA into the circulation could complicate identification of ctDNA (Ignatiadis *et al*., 2015). Technically, the surge of wild‐type cfDNA would increase the sensitivity and specificity requirements for the ctDNA detection method. Therefore, awareness of wild‐type cfDNA fluctuations in ctDNA detection settings is paramount, and care should be taken to avoid increased cfDNA levels.

The half‐life of cfDNA is approximately 2 h (Diehl *et al*., 2008). Still, cfDNA concentrations have been shown to be elevated several days and even weeks after trauma, with the time to return to pretraumatic cfDNA levels varying according to trauma severity (Lam *et al*., 2003). Increases in trauma‐induced cfDNA concentration and their implication in ctDNA detection have not been described for cancer surgery. Using ctDNA to guide the postsurgical treatment decisions for cancer patients has been proposed in multiple studies showing an association between postoperative ctDNA and increased risk of disease recurrence (Christensen *et al*., 2019; Reinert *et al*., 2019; Schøler *et al*., 2017; Tie *et al*., 2016). Other studies have shown that to be effective, adjuvant treatment should be initiated shortly after surgery (Biagi *et al*., 2011; Klein *et al*., 2015). If surgery is associated with elevated cfDNA levels, it is important to describe the time it takes to return to normal cfDNA levels, to optimize blood sampling time for postsurgical treatment decisions.

Cell‐free DNA is released mostly through apoptosis of cells, yielding short, nucleosomal DNA fragments of approx. 166 bp, corresponding to a chromatosome (nucleosome and linker histone) (Jahr *et al*., 2001). Whether trauma‐induced cfDNA is similarly fragmented has not yet been reported. If trauma‐induced cfDNA is indeed less fragmented, one possible method to avoid trauma‐induced cfDNA could be to separate it from standard nucleosomal cfDNA based on size.

In this study, we use plasma from patients with colorectal cancer or muscle‐invasive bladder cancer—treated with curative intent surgery—to monitor trauma‐induced changes in cfDNA levels for up to 6 weeks after cancer surgery. Further, we aimed to characterize the degree of fragmentation of trauma‐induced cfDNA to potentially discern trauma‐induced cfDNA from regular cfDNA and ctDNA. Finally, we aimed to investigate whether trauma‐induced cfDNA impacts the ability to detect ctDNA using tumor‐informed ultradeep plasma cell‐free sequencing.

## 2 Methods

### 2.1 Patient inclusion

A total of 453 patients undergoing elective surgery for stage I–III colorectal cancer were recruited at the Surgical Departments of Aarhus University Hospital, Randers Hospital, Herning Hospital, Viborg Hospital, Aalborg University Hospital, Odense University Hospital, and Slagelse Hospital in Denmark. Patients with colorectal cancer were enrolled between 2014 and 2019. Further, a total of 47 patients diagnosed with muscle‐invasive bladder cancer and treated with radical cystectomy were recruited at Aarhus University Hospital between 2013 and 2017.

The study was approved by the Committees on Biomedical Research Ethics in the Central Region of Denmark (1‐16‐02‐453‐14 and #1706501) and was performed in accordance with the Declaration of Helsinki. All participants provided written informed consent.

### 2.2 Sample collection

Pre‐ and postoperative blood samples were drawn for each patient. For patients receiving neoadjuvant treatment, the preoperative blood sample was collected before treatment start. Blood samples were collected in K2‐EDTA 10‐mL tubes (Cat: 366643; Becton Dickinson, Franklin Lakes, NJ, USA) at participating hospitals. All samples were processed within 2 h of collection by centrifugation of the blood at room temperature; first for 10 min at 3000 ***g*** and followed by centrifugation of plasma for 10 min at 3000 ***g*** for the samples from patients with colorectal cancer. Aliquots of 5 mL plasma were transferred to cryotubes and stored at −80 °C.

### 2.3 cfDNA isolation and quantification

DNA was purified from 8 mL of plasma using the QIAamp Circulating Nucleic Acids Kit (Cat: 55114; Qiagen, Hilden, Germany), and eluted in a volume of 50–60 µL according to the manufacturer's protocol. In samples from patients with colorectal cancer, the number of cfDNA copies in each plasma sample was determined by digital droplet polymerase chain reaction (ddPCR; Bio‐Rad Laboratories, Hercules, CA, USA) assays specific to two highly conserved regions on Chr3 and Chr7, as described previously (Reinert *et al*., 2016). In brief, the ddPCR was carried out in a 20 µL reaction volume with 2 µL input of DNA eluate as template. The primer and probe sequences, amplicon size, and PCR amplification protocol of all used ddPCR assays are provided in Table S1. PCR mixture was made according to the manufacturer's instructions (Bio‐Rad), and droplets were formed on the Automated Droplet Generator (Bio‐Rad). PCRs were run on the S1000 Thermal Cycler (Bio‐Rad), and droplets were analyzed on a QX100™ Droplet Reader (Bio‐Rad). The mean signal from Chr3 and Chr7 was used to calculate the cfDNA concentration in the plasma eluate. Prior to DNA purification, a fixed number of copies of a PCR fragment from the soybean gene encoding the DNA binding protein CPP1 were spiked into the plasma samples. The number of remaining CPP1 copies was quantified in the purified DNA using a CPP1‐specific ddPCR assay, and used as an internal control for purification efficiency (Pallisgaard *et al*., 2015). Quantification of the ddPCR readout was done through the quantasoft™ software (v1.7.4; Bio‐Rad). The DNA concentration in the plasma eluate was corrected for purification efficiency.

In samples from patients with muscle‐invasive bladder cancer, cfDNA was quantified by the Quant‐iT High‐Sensitivity dsDNA Assay Kit (Cat: Q33120; Invitrogen, Carlsbad, CA, USA), as previously described (Christensen *et al*., 2019).

### 2.4 cfDNA size characterization

Using dual‐sided selection with SPRI beads (Cat: A63882, Agencourt, Beckman Coulter, Brea, CA, USA), cfDNA was separated in two fractions with short (< 1 kb) and long (> 1 kb) DNA fragments, respectively. The size‐separated cfDNA was profiled on a LabChip GX (PerkinElmer, Waltham, MA, USA) and quantified using the high‐sensitivity Quant‐iT dsDNA assay (Invitrogen).

### 2.5 ctDNA measurements

For a subset of patients in this study, we have previously measured and reported postoperative ctDNA quantifications in both colorectal cancer (Reinert *et al*., 2019) (*n* = 91) and muscle‐invasive bladder cancer (Christensen *et al*., 2019) (*n* = 47). From these, patients with radiologically confirmed relapse were selected (*n* = 25). Analysis of ctDNA masked by cfDNA was performed for patients, who were ctDNA‐negative immediately after surgery and became ctDNA‐positive before month 6 (*n* = 8).

### 2.6 Statistical analysis

Postoperative cfDNA concentrations were normalized to preoperative cfDNA concentrations for each patient. Dependent on the day of blood collection, the postoperative data were binned in 1‐week intervals. To achieve statistical power in the total cfDNA analyses, weeks with less than five samples were binned with the following week. A paired Wilcoxon signed rank test was used to compare postoperative and preoperative cfDNA levels for total, long, and short cfDNA fragments. For the patients with colorectal cancer, this comparison was also stratified for UICC stages (I, II and III), tumor locations (colon and rectum), old (> mean age) and young (≤ mean age) patients, and sex (male and female). All statistical analyses were performed using r (v.3.4, R Foundation for Statistical Computing, Vienna, Austria).

## 3 Results

In this study, 453 patients with colorectal cancer (stage I–III) and 47 patients with muscle‐invasive bladder cancer undergoing curatively intended surgery were included (Table 1 and Fig. S1). Each patient had a preoperative and a postoperative blood sample collected. The preoperative blood was collected up to 287 days before surgery (colorectal cancer, median 3 days before, range 0–22 days; muscle‐invasive bladder cancer, median 123 days before, range 49–287 days). The postoperative sample was collected between day 1 and day 42 after surgery (colorectal cancer, median day 15, range 1–41; muscle‐invasive bladder cancer, median day 21, range 6–42). To avoid adjuvant chemotherapy confounding the assessment of the postoperative cfDNA levels, patients with blood collected during adjuvant chemotherapy were excluded, while patients with blood sample collected prior to initiation of adjuvant chemotherapy were not excluded. In total, 436 patients with colorectal cancer and 47 patients with muscle‐invasive bladder cancer were analyzed. Thirty‐one patients with muscle‐invasive bladder cancer had two postoperative samples collected. These were analyzed as independent samples. The blood collection time‐points for each patient are shown in Figs [Link], [Link].

**Table 1:** Patient characteristics and demographics of eligible patients.

	Colorectal cancer	Bladder cancer
Age (years), median (range)	70 (41–91)	65 (47–80)
Sex, *n* (%)
Male	246 (56)	36 (77)
Female	190 (44)	11 (23)
Pathological UICC stage, *n* (%)
I	95 (22)	33 (70)
II	176 (40)	1 (2)
III	165 (38)	4 (9)
IV	0 (0)	9 (19)
Tumor location, *n* (%)
Colon	333 (76)	–
Rectum	103 (24)	–

### 3.1 cfDNA levels increase following surgical trauma

**Fig. 1:**
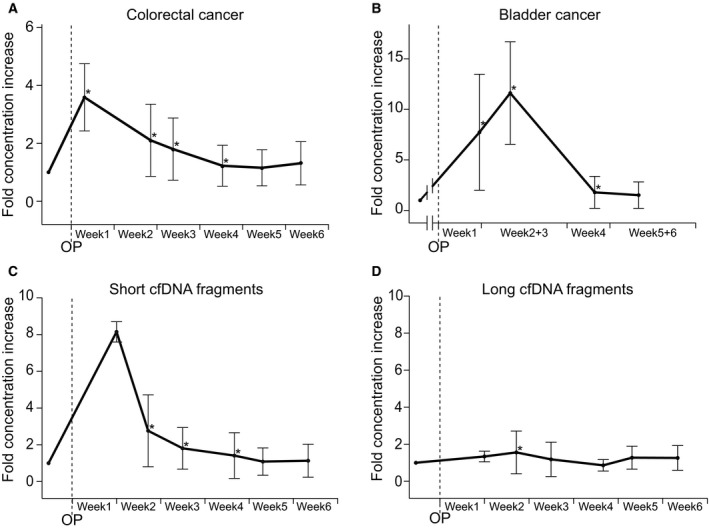
Changes in cfDNA concentration after surgery. The median changes in cfDNA concentration relative to preoperative levels are shown for: (A) total cfDNA in samples from CRC patients (*N* = 436); (B) total cfDNA in samples from MIBC patients (*N* = 47); (C) short cfDNA fragments (*N* = 91); (D) long cfDNA fragments (*N* = 91) at different times after surgery. Plasma samples were organized in weekly bins based on the postoperative day the blood was collected. To account for varying interpatient baseline cfDNA concentrations, the postoperative cfDNA levels were normalized to the preoperative level for each patient. Error bars indicate median absolute deviation. Time after surgery depicts the mean time for all samples within that time bin. Lines drawn between data points are meant as guides and do not signify longitudinal samples. Statistical significance (*P* ≤ 0.05) in fold concentration change calculated by the Wilcoxon signed rank test is indicated by ‘*’. OP, time of operation.

The cfDNA level was quantified pre‐ and postoperatively for each patient. The postoperative cfDNA level was on average threefold (*P* ≤ 0.0001) higher than the preoperative level in samples from patients with colorectal cancer and eightfold (*P* ≤ 0.0001) higher in samples from patients with muscle‐invasive bladder cancer. To investigate the duration of the cfDNA increase, the postoperative cfDNA levels were plotted as a function of time (Fig. 1A,B, Fig. S4). In samples from patients with colorectal cancer, the largest cfDNA level increase was observed during the first week after the trauma (median 3.6‐fold increase, mean: 4.0, 95% CI 2.90–5.37, *P* = 0.0005), and levels gradually declined over the next 3 weeks, before returning to pretrauma levels in week 5 (Table 2 and Table S2). The fraction of samples with a ≥ 3‐fold increase was 67% in week 1 and gradually declined to 13% in week 4, and 0% in week 5 (Table 2). The postoperative cfDNA increase occurred independent of sex, age, UICC stage, and tumor location (Table S3).

**Table 2:** Postoperative change in cfDNA concentration relative to the preoperative level. MAD, median absolute deviation.

	*n* ^a^	Median ± MAD	Mean	95% CI	*P*‐value^b^	> Mean^c^
Colorectal cancer
Week 1	12	3.6 ± 1.16	4	2.91–5.28	**0.0005**	67%
Week 2	156	2.11 ± 1.25	3.89	3.01–4.97	**< 0.0001**	33%
Week 3	200	1.81 ± 1.07	2.81	2.21–3.66	**< 0.0001**	23%
Week 4	48	1.23 ± 0.71	2.66	1.43–4.81	**0.0005**	13%
Week 5	13	1.16 ± 0.62	1.21	0.92–1.51	0.2734	0%
Week 6	7	1.32 ± 0.75	1.43	0.87–1.99	0.2969	0%
Bladder cancer
Week 1	32	7.73 ± 5.74	10.26	7.75–13.13	**< 0.0001**	47%
Week 2 + 3	7	11.62 ± 5.07	13.13	7.8–20.23	**0.0156**	71%
Week 4	23	1.79 ± 1.58	8.44	2.18–19.97	**< 0.0001**	13%
Week 5 + 6	16	1.51 ± 1.31	1.92	1.25–2.7	0.0577	0%

^a^ *n* denotes the number of blood samples taken in the given time period.

^b^ Statistically significant *P*‐values (*P* ≤ 0.05) marked in bold.

^c^ Proportion of samples with cfDNA increase > mean increase of all postsurgical samples (colorectal cancer: mean = 3, muscle‐invasive bladder cancer: mean = 8).

In the samples from patients with muscle‐invasive bladder cancer, cfDNA levels increased during week 1 with a median 7.7‐fold increase and reached the highest levels during weeks 2 and 3 with a median 11.6‐fold increase (mean: 13.1, 95% CI 7.6–19.6, *P* = 0.0156). From then, the level gradually declined before returning to preoperative levels in weeks 5 and 6. The fraction of samples with a ≥ 8‐fold increase was 47% in week 1, increased to 71% in weeks 2 and 3, and declined to 13% in week 4 and 0% in weeks 5 and 6 (Table 2).

### 3.2 Size profiling of colorectal cancer surgical trauma‐induced cfDNA

Cell‐free DNA size fractionation into short (peak size ~ 166 base pairs) and long (smear size > 1 kb) cfDNA fragments was performed on matched pre‐ and postoperative plasma samples from 91 patients with colorectal cancer. Examples of size profiles are shown in Fig. S5. The short cfDNA fragment level increased to a median 8.2‐fold already in week 1 (mean: 8.2, 95% CI 7.78–8.52, *P* = 0.5) and gradually decreased in weeks 2 (median: 2.8, mean: 4.0, 95% CI 2.81–5.39, *P* < 0.0001), 3 (median: 1.8, mean: 2.58, 95% CI 1.64–3.71, *P* = 0.0006), and 4 (median: 1.4, mean: 2.8, 95% CI 1.63–4.28, *P* = 0.0094) before reaching the preoperative level in week 5 (median: 1.1, mean: 1.91, 95% CI 1.91–3.9, *P = *1) (Fig. 1C, Table S4). By contrast, the level of the long fragments hardly changed (Fig. 1D). The long cfDNA fragment levels were briefly elevated at week 2 with a median increase of 1.6‐fold (mean: 3.0, 95% CI 1.95–4.17, *P* < 0.0001). In all other weeks, the postoperative levels were not significantly different from the preoperative levels (Table S4).

### 3.3 Trauma‐induced cfDNA and the ability to detect ctDNA

**Fig. 2:**
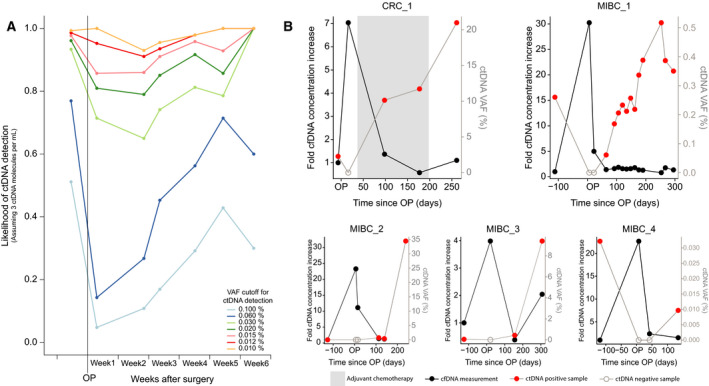
ctDNA detection in samples with elevated cfDNA levels. (A) Theoretical likelihood of ctDNA detection in CRC samples (*N* = 436), assuming three ctDNA molecules per millilitre of plasma. The likelihood of ctDNA detection is simulated based on different minimum detectable VAF cutoffs. Plasma samples were sorted in weekly bins based on the postoperative day the blood was collected. (B) Fold increase in cfDNA concentration and absolute ctDNA VAF in CRC (*N* = 1) and MIBC (*N* = 4) patients over time. All analyzed postoperative plasma samples until radiological detection of disease recurrence is shown.

With increasing levels of trauma‐induced cfDNA, the requirements to the technical sensitivity and specificity also increase for any approach intended for ctDNA detection. The likelihood of ctDNA detection varies with a given technique's minimal detectable ctDNA variant allele frequency (VAF). In our previous studies of colorectal cancer, we observed a median of three tumor molecules per millilitre of plasma immediately after surgery in ctDNA‐positive samples (Reinert *et al*., 2019). Figure 2A shows the proportion of samples in our colorectal cancer cohort, where ctDNA detection would be theoretically possible at different VAF cutoffs for detection of ctDNA, when assuming the presence of three tumor molecules per millilitre of plasma. ctDNA detection techniques that are unable to detect ctDNA with a VAF less than 0.06% would theoretically detect ctDNA in less than 30% of samples collected at week 2 from patients with colorectal cancer. The same sensitivity would detect 70% of samples in week 5. To achieve detection of > 90% of samples in every week, the minimum detectable ctDNA VAF has to be lower than 0.012%.

### 3.4 Elevated cfDNA levels hamper ctDNA detection

Using ctDNA data from our previously published studies (Christensen *et al*., 2019; Reinert et al., 2019), we investigated the impact of trauma‐induced DNA release on the ability to detect ctDNA postoperatively in patients with colorectal cancer and patients with muscle‐invasive bladder cancer. In these studies, longitudinally collected plasma samples were analyzed for ctDNA by ultradeep targeted plasma DNA sequencing. A total of 25 patients had radiologically confirmed relapse diagnosed during follow‐up and were thus candidates for being ctDNA‐positive. In eight (32%) patients, ctDNA was detected during the trauma‐induced DNA period (weeks 1–4). Next, we investigated whether trauma‐induced cfDNA hampered ctDNA detection in the remaining 17 patients with metastatic relapse. Six had no plasma samples collected after week 4 and were excluded from further analysis. In three patients, no ctDNA was detected in the two to three samples collected before 6 months after surgery. In these patients, it is most likely that the ctDNA levels immediately after surgery would be too low for any ctDNA detection technique to detect regardless of cfDNA concentration. Therefore, these patients were also excluded from further analysis. For the remaining eight patients, ctDNA was detected between week 4 and month 6. Overlaying the measured cfDNA and ctDNA levels indicate that in five out of eight patients, the trauma‐induced cfDNA increase probably hindered ctDNA detection (Fig. 2B, Fig. S6). For these patients, ctDNA was detected before surgery and an increase in cfDNA levels was observed during the first month after surgery, where no ctDNA was detected. Following samples tested positive for ctDNA, and cfDNA levels had decreased to preoperative levels. ctDNA detection persisted until subsequent radiological relapse. In the remaining three out of eight patients, ctDNA was not detected immediately after cfDNA levels had decreased to baseline levels (Fig. S7).

## 4 Discussion

In this study, we aimed to describe how surgically induced trauma affects postoperative cfDNA levels and in turn impacts postoperative ctDNA detection. We also sought to characterize whether trauma‐induced cfDNA was of similar size to normal cfDNA, to evaluate whether simple size selection could eliminate the trauma‐induced cfDNA.

We demonstrate that the total cfDNA level increases immediately after surgery and that the elevation persists up to 4 weeks after surgery. Interestingly, the mean increase in cfDNA levels was higher in patients with muscle‐invasive bladder cancer (eightfold increase) than patients with colorectal cancer (threefold increase). This could indicate that cystectomy is associated with a more severe trauma than a colon or rectum resection.

Studying patients with cancer undergoing surgery, we had the unique ability to study paired pre‐ and postoperative samples to provide direct documentation for elevated cfDNA levels postoperatively. Most trauma‐related cfDNA studies have been carried out in acute trauma patients, where baseline samples could not be obtained (Gögenur *et al*., 2017; Jackson Chornenki *et al*., 2019; Ren *et al*., 2013). In these cases, healthy controls were used as a baseline to provide indirect evidence of posttraumatic cfDNA changes. Some studies using transplant patients have compared pre‐ and postoperative cfDNA levels (Prakash *et al*., 2017; Qi *et al*., 2016), but are complicated by the introduction of a foreign organ (transplant), and thus not suitable to determine the contribution of the surgical trauma to the elevation in cfDNA.

Our data further document how trauma‐induced DNA release could affect the ability to detect ctDNA. We show that a ctDNA detection method with low sensitivity (such as 0.06% VAF) would have a higher likelihood of detecting ctDNA during week 5 than week 2. A minimum detectable VAF of 0.012% is required to detect ctDNA in > 90% of samples in our colorectal cancer cohort, regardless of sample timing. The minimum detectable VAF for most ctDNA detection techniques ranges from 0.01% to 1% (Elazezy and Joosse, 2018). As only few techniques have a sensitivity of 0.01% VAF or below, avoiding contamination from trauma‐induced cfDNA would be ideal in all settings.

Using previously published ultradeep targeted plasma DNA sequencing data, we investigated the effect of trauma‐induced cfDNA on ctDNA detection. In blood samples collected within week 1–4, ctDNA was missed in samples from 32% (*n* = 8) of patients that experienced molecular relapse within 6 months after surgery and subsequently experienced radiological relapse. As ctDNA was detected within 6 months, the tracked mutation was present in the residual tumor cells. That ctDNA was missed in the sample taken immediately after surgery was likely due to the level of ctDNA relative to cfDNA being too low. In 63% of these patients (5/8), the ctDNA was potentially not detected due to massive release of trauma‐induced cfDNA. In all five cases, ctDNA was readily detected in blood samples collected after the cfDNA level had returned to preoperative levels. It is also possible that ctDNA was not detected, as the absolute level of ctDNA was inadequate. After all, ctDNA levels are expected to be low after surgery, where the tumor burden is at a minimum. However, delaying collection of the postoperative blood samples to week 5 or later, when cfDNA levels are expected to have returned to normal, would decrease the likelihood of diluting ctDNA with large amounts of trauma‐induced cfDNA. This would eliminate one of the causes of a low ctDNA‐to‐cfDNA ratio. Ideally, these findings should be validated in a large cohort of patients with blood samples drawn 2 and 5 weeks after surgery, so they can be compared directly.

Multiple studies have suggested the use of ctDNA as a guide for adjuvant treatment decisions in colorectal cancer, due to the increased risk of relapse associated with ctDNA presence (Reinert *et al*., 2019; Tie *et al*., 2016). When treating colorectal cancer with adjuvant chemotherapy, it is recommended to initiate the regimen as soon as the patient has recovered from surgery. If ctDNA is to be used as a guide for adjuvant treatment, it is critical that the time‐point for blood collection allows time for ctDNA analysis, before adjuvant treatment is to be initiated. However, with contamination of trauma‐induced cfDNA up to 4 weeks after surgery, the window of opportunity diminishes for collecting blood and performing ctDNA analysis without trauma‐induced cfDNA. The turnaround times reported for ctDNA analysis vary from days (Sacher *et al*., 2016) to weeks (Schwaederle *et al*., 2017) for different ctDNA detection methods. The time needed for ctDNA analysis should therefore be factored in when considering the timing of blood draws in the clinical setting, and when choosing the ctDNA analysis method. An option could be to draw blood samples as early as possible, for example, 2 weeks after surgery, and again 5 weeks after surgery. If ctDNA can be detected in the early blood sample, adjuvant treatment can be initiated sooner. If not, the later blood sample could provide a better opportunity for ctDNA detection, as VAFs would be higher. This strategy would, however, likely increase analysis costs, as the majority of patients do not have residual disease and are expected to be ctDNA‐negative both 2 and 5 weeks after surgery. Furthermore, for some methods, for example, sequencing‐based methods, the increased cfDNA levels shortly after surgery will cause increased analysis costs, as it will be necessary to sequence deeper (sequence all the contaminating wild‐type trauma DNA) to find the tumor DNA. An increased sequencing depth may also affect the noise of the method and consequently its specificity, sensitivity and clinical utility. Also, sequencing methods capping the DNA input may be subject to substantial subsampling errors, when samples contain large amounts of wild‐type DNA.

We hypothesized that if the trauma‐induced cfDNA could be separated from the rest of the cfDNA, then the stricter requirements to the ctDNA detection methods could be averted. To this end, we examined the size profile of pre‐ and postoperative blood samples, to investigate whether removal of long cfDNA fragments prior to ctDNA analysis would remove the trauma‐induced cfDNA. An increase was observed in the concentration of shorter nucleosomal cfDNA fragments, mimicking the pattern observed for the total cfDNA fraction. The concentration of longer fragments remained reasonably steady after surgery. Accordingly, the postoperative increase in cfDNA was caused by trauma‐related release of DNA fragments of similar size to normal cfDNA and ctDNA. Consequently, removal of long DNA fragments is not likely to mitigate the problems introduced by trauma‐induced cfDNA. The size similarity between ordinary and trauma‐induced cfDNA indicates a common mechanism of fragmentation, most likely apoptosis.

There are some limitations to this study. Ideally, longitudinal samples would make comparisons of cfDNA dynamics easier. However, the ability to normalize postoperative cfDNA levels to preoperative cfDNA levels for each patient should largely overcome this limitation. The lower number of samples drawn during the first, fifth, and sixth week after surgery compared to the second, third, and fourth week also makes it difficult to pinpoint the exact time of cfDNA concentration returning to normal. On the other hand, there seems to be good agreement between the data from the colorectal cancer and muscle‐invasive bladder cancer cohorts. Furthermore, our results are in agreement with a previous report on a smaller cohort of lung cancer patients (Hu *et al*., 2017).

## 5 Conclusions

Surgical trauma causes increased cfDNA levels for up to 4 weeks. Hence, when drawing postoperative blood samples for ctDNA detection, the timing of blood sampling should be carefully considered, as later sampling considerably reduces the issue with trauma‐induced wild‐type cfDNA contamination. This strategy will be feasible and beneficial in all situations where delayed blood sampling does not prohibit completion of the ctDNA analysis in due time before postsurgical treatment decisions.

## Conflict of interest

Sethi and Zimmermann are employees of Natera, Inc. and own stock, or options to stock, in the company. The remaining authors have nothing to disclose.

## Author contributions

TVH, TR, and CLA designed the experiments. TVH wrote the manuscript with revisions by all coauthors. TVH, TR, and CLA gathered all data on colorectal cancer patients. EC, KB‐D, and LD provided all data on bladder cancer patients. HS and BGZ further provided all data on size separations. IG and MG provided feedback on the statistical analyses and the manuscript, and helped procure a subset of the blood samples, and the remaining blood samples were collected through the IMPROVE study group. All authors, including the IMPROVE study group, approved of the manuscript before submission.

## Supporting information


**Fig. S1.** Flow of patient exclusion and inclusion in subanalyses.

**Fig. S2.** Overview of blood sample draws from all included colorectal cancer patients (N = 436).

**Fig. S3.** Overview of blood sample draws from all included muscle‐invasive bladder cancer patients (N = 47).

**Fig. S4.** Changes in cfDNA concentration after surgery.

**Fig. S5.** Representative size profile traces from five different plasma samples.

**Fig. S6.** Absolute cfDNA and ctDNA concentration (GE/mL) in CRC and MIBC patients over time.

**Fig. S7.** Fold increase in cfDNA concentration and absolute ctDNA VAF in CRC and MIBC patients over time.

**Table S1.** Primer and TaqMan probe sequences and amplification protocols for cfDNA quantification.
**Table S2.** Total cfDNA concentration in Genome Equivalents pr mL plasma.
**Table S3.** Postoperative change in cfDNA concentration for CRC patients stratified for UICC stage, tumor localization, age and sex.
**Table S4.** Postoperative change in cfDNA concentration in short and long cfDNA fragments.

## Data Availability

Data can be made available upon reasonable request.
