# Supplementary figures and images for: The effect of surgical trauma on circulating free DNA levels in cancer patients—implications for studies of circulating tumor DNA
Source: Mol Oncol. 2020 Jun 16;14(8):1670–9. doi: 10.1002/1878-0261.12729 (PMC7400779; doi:10.1002/1878-0261.12729)

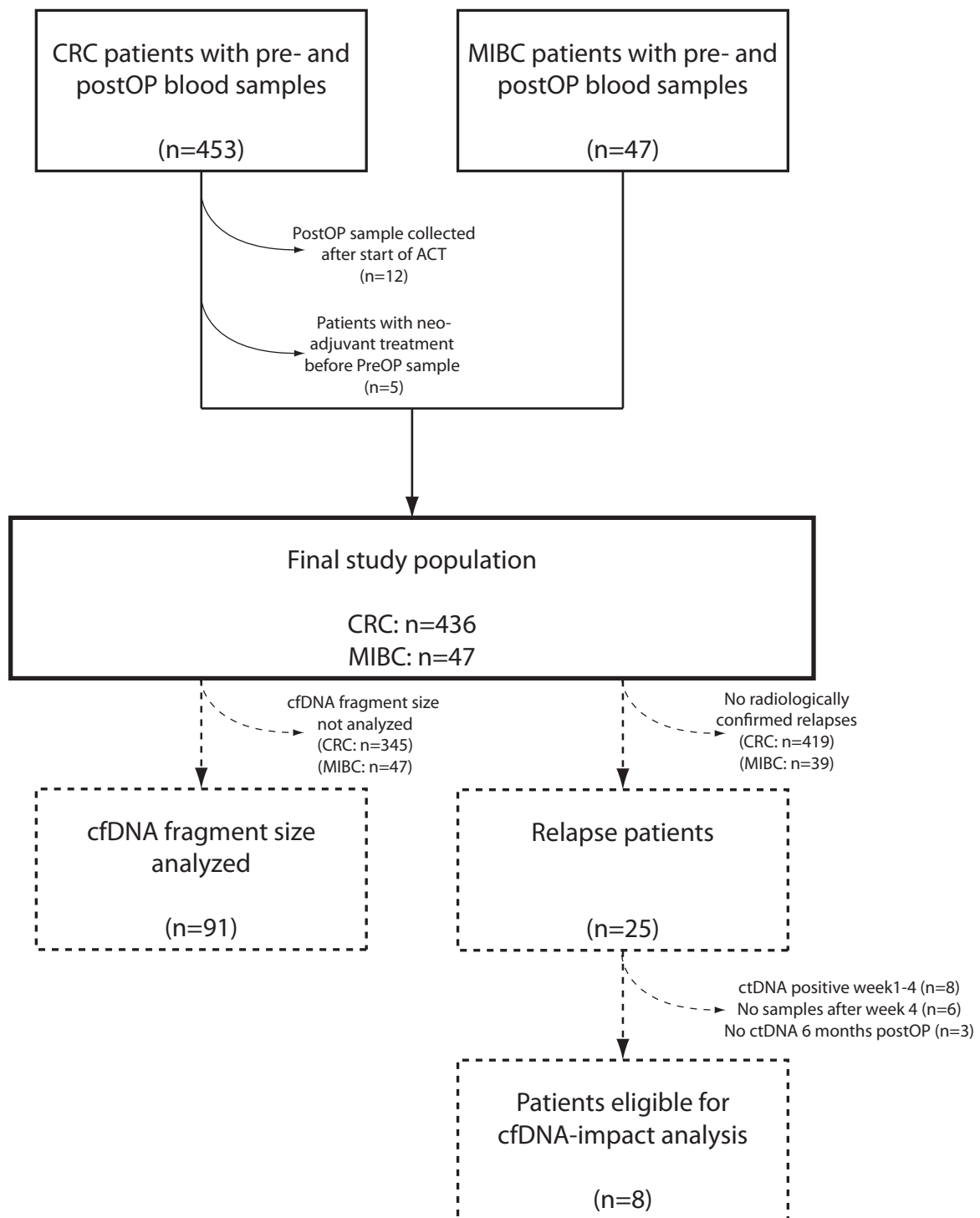

**Supplementary Figure 1 – Flow of patient exclusion and inclusion in sub-analyses.**

Supplement: Supplementary file 1 — Fig. S1. Flow of patient exclusion and inclusion in subanalyses. [file MOL2-14-1670-s001.pdf]
