# Supplementary material for: The effect of surgical trauma on circulating free DNA levels in cancer patients—implications for studies of circulating tumor DNA
Source: Mol Oncol. 2020 Jun 16;14(8):1670–9. doi: 10.1002/1878-0261.12729 (PMC7400779; doi:10.1002/1878-0261.12729)
Supplement: Supplementary file 3 — Fig. S3. Overview of blood sample draws from all included muscle‐invasive bladder cancer patients (N = 47). [file MOL2-14-1670-s003.pdf]

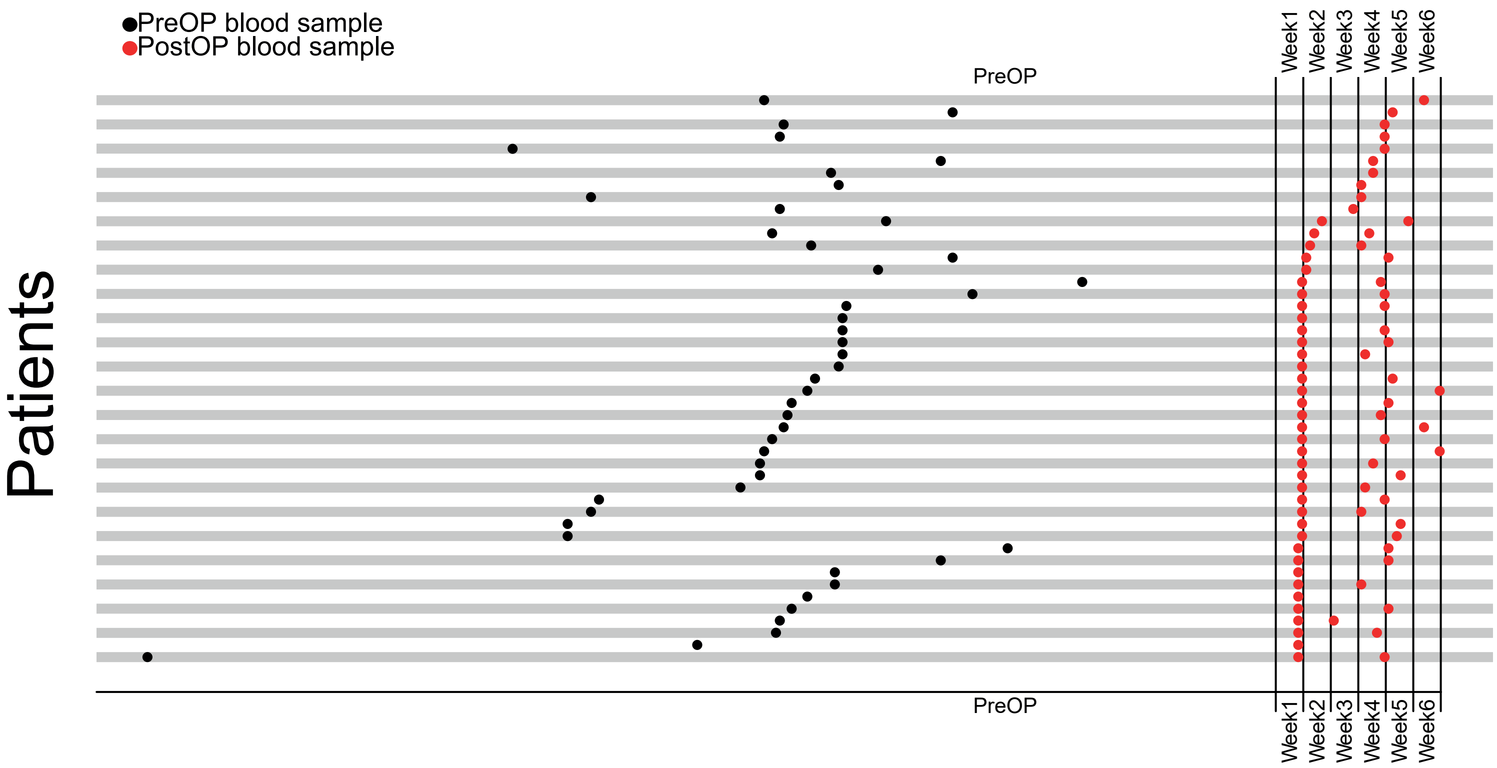

Supplementary Figure 3 – Overview of blood sample draws from all included muscle-invasive bladder cancer patients (N=47).
