# Supplementary material for: The effect of surgical trauma on circulating free DNA levels in cancer patients—implications for studies of circulating tumor DNA
Source: Mol Oncol. 2020 Jun 16;14(8):1670–9. doi: 10.1002/1878-0261.12729 (PMC7400779; doi:10.1002/1878-0261.12729)
Supplement: Supplementary file 4 — Fig. S4. Changes in cfDNA concentration after surgery. [file MOL2-14-1670-s004.pdf]

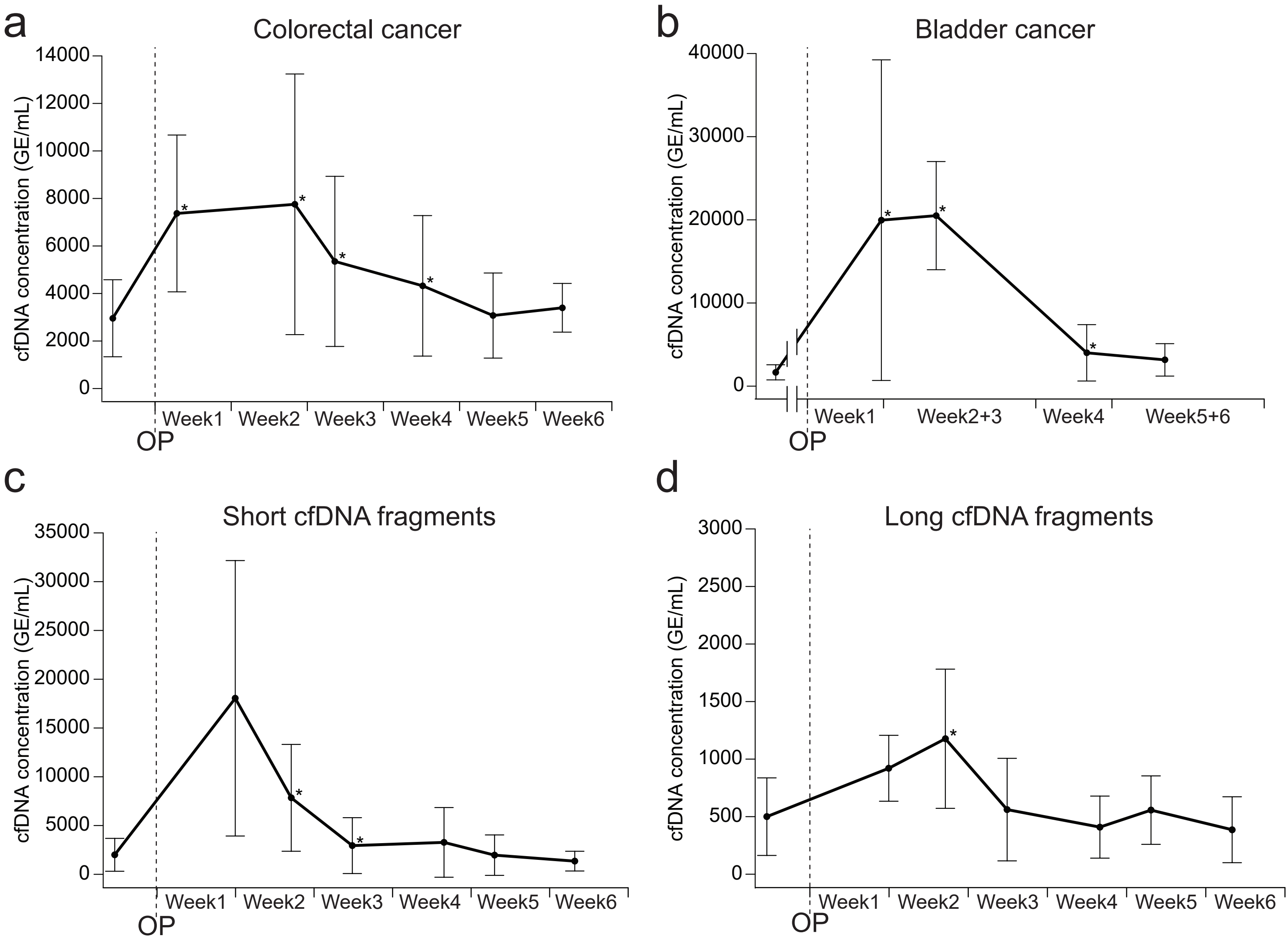

Supplementary Figure 4 – Changes in cfDNA concentration after surgery. The median changes in cfDNA concentration are shown for: a) total cfDNA in samples from CRC patients (N=436); b) total cfDNA in samples from MIBC patients (N=47); c) Short cfDNA fragments (N=91); d) long cfDNA fragments (N=91) at different times after surgery. Plasma samples were organized in weekly bins based on the postoperative day the blood was collected. Error bars indicate Median Absolute Deviation (MAD). Time after surgery depicts the mean time for all samples within that time bin. Lines drawn between data points are meant as guides and do not signify longitudinal samples. Statistical significance (P≤0.05) in fold concentration change calcula-ted by the Wilcoxon Signed Rank test is indicated by "*". OP = Time of operation.
