# Supplementary material for: The effect of surgical trauma on circulating free DNA levels in cancer patients—implications for studies of circulating tumor DNA
Source: Mol Oncol. 2020 Jun 16;14(8):1670–9. doi: 10.1002/1878-0261.12729 (PMC7400779; doi:10.1002/1878-0261.12729)
Supplement: Supplementary file 5 — Fig. S5. Representative size profile traces from five different plasma samples. [file MOL2-14-1670-s005.pdf]

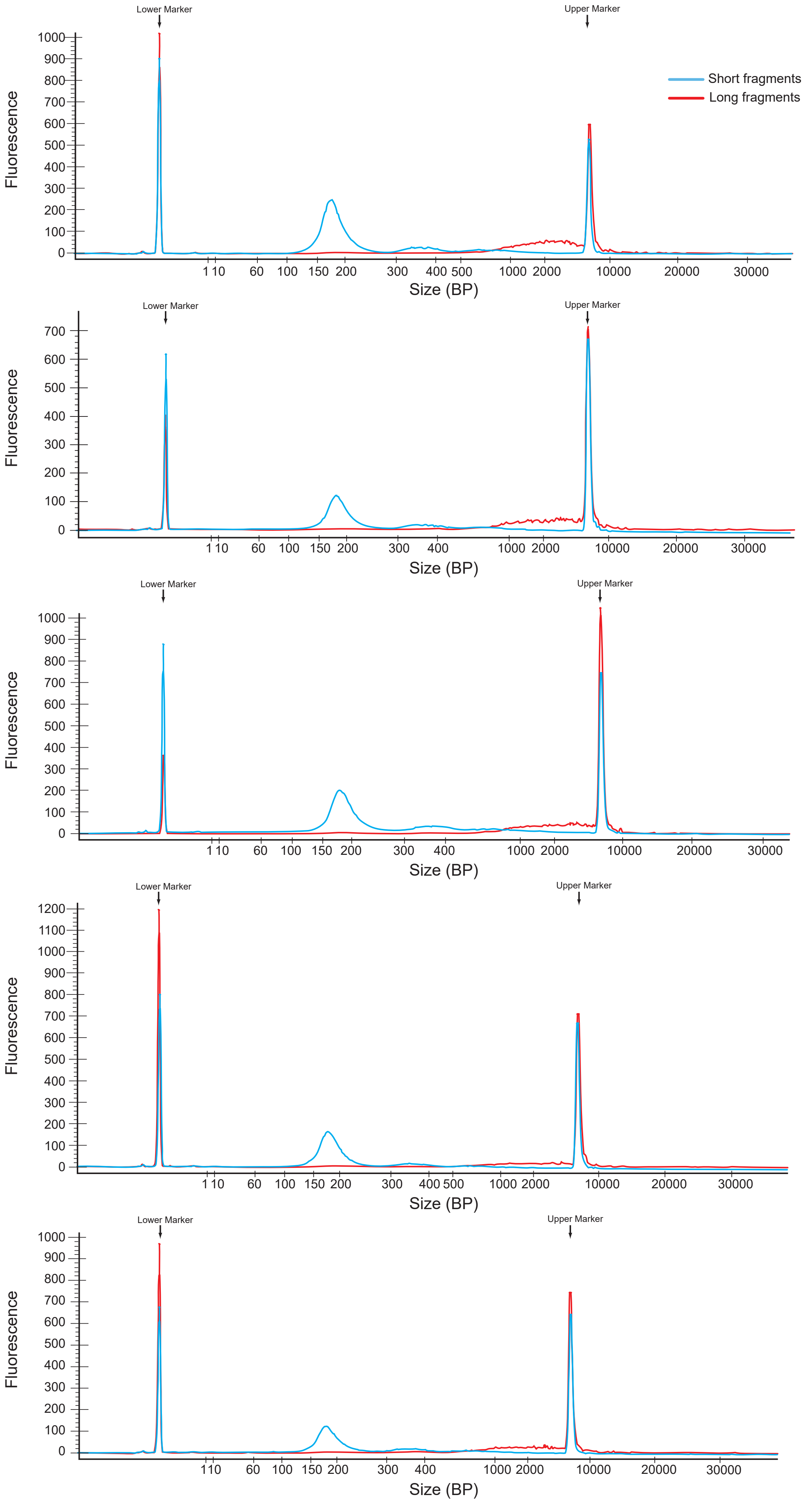

Supplementary Figure 5 – Representative size profile traces from five different plasma samples.
