# Supplementary material for: The effect of surgical trauma on circulating free DNA levels in cancer patients—implications for studies of circulating tumor DNA
Source: Mol Oncol. 2020 Jun 16;14(8):1670–9. doi: 10.1002/1878-0261.12729 (PMC7400779; doi:10.1002/1878-0261.12729)
Supplement: Supplementary file 6 — Fig. S6. Absolute cfDNA and ctDNA concentration (GE/mL) in CRC and MIBC patients over time. [file MOL2-14-1670-s006.pdf]

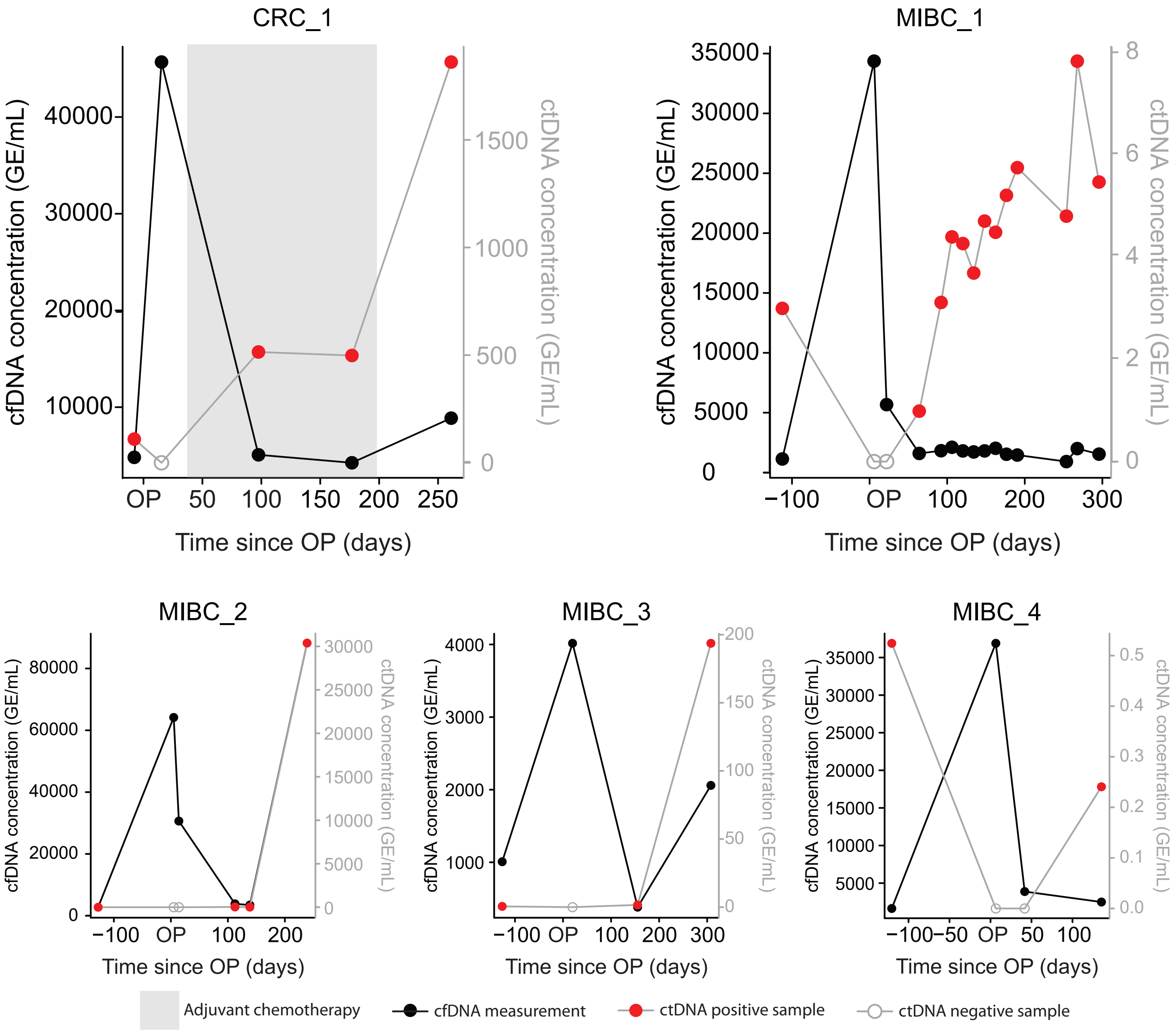


**Supplementary Figure 6 - Absolute cfDNA and ctDNA concentration (GE/mL) in CRC and MIBC patients over time.** All analyzed postoperative plasma samples until radiological detection of disease recurrence are shown.
