# Supplementary material for: The effect of surgical trauma on circulating free DNA levels in cancer patients—implications for studies of circulating tumor DNA
Source: Mol Oncol. 2020 Jun 16;14(8):1670–9. doi: 10.1002/1878-0261.12729 (PMC7400779; doi:10.1002/1878-0261.12729)
Supplement: Supplementary file 7 — Fig. S7. Fold increase in cfDNA concentration and absolute ctDNA VAF in CRC and MIBC patients over time. [file MOL2-14-1670-s007.pdf]

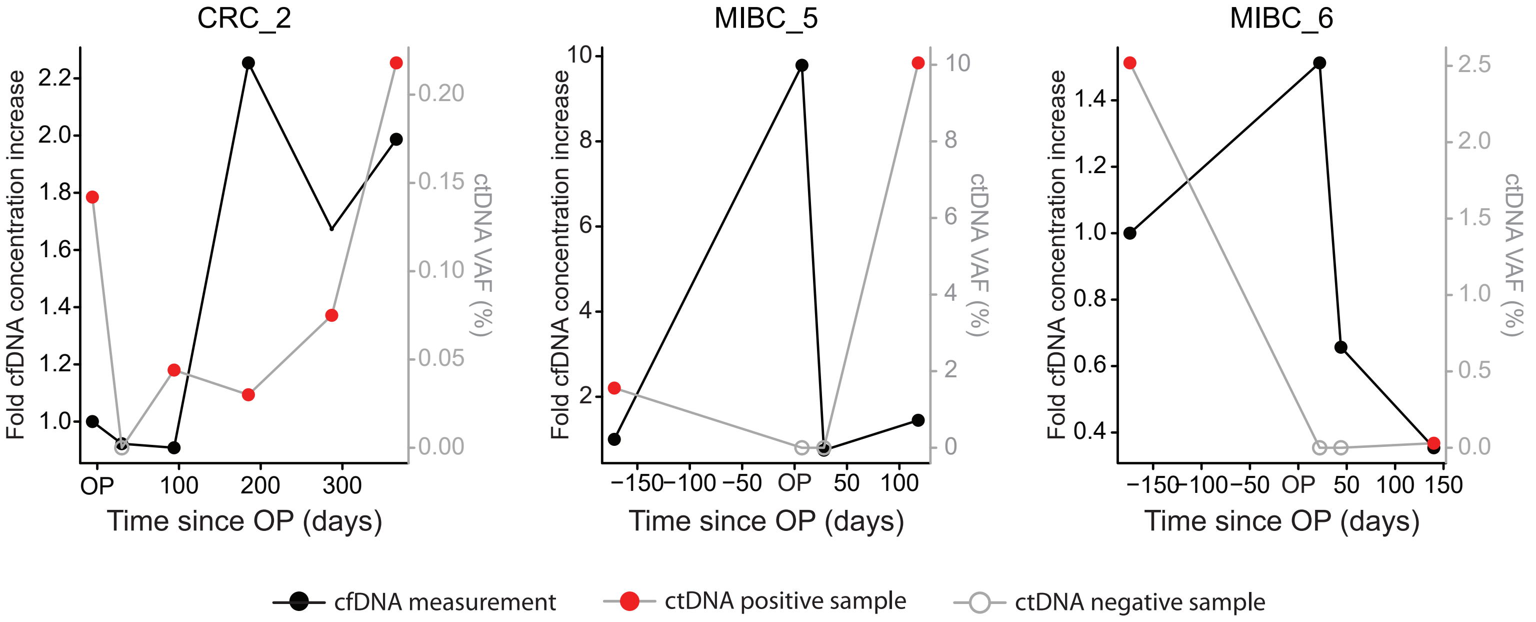

Supplementary Figure 7 – Fold increase in cfDNA concentration and absolute ctDNA VAF in CRC and MIBC patients over time. Shown for patients, where ctDNA was likely not masked by trauma-induced cfDNA. Likely, the tumor burden was extremely low postoperatively in these patients, and were therefore not detected immediately after surgery. The subsequent detection reflects increased tumor burden rather than decreased cfDNA levels. All analyzed postoperative plasma samples until radiological detection of disease recurrence are shown.
