# Supplementary material for: The effect of surgical trauma on circulating free DNA levels in cancer patients—implications for studies of circulating tumor DNA
Source: Mol Oncol. 2020 Jun 16;14(8):1670–9. doi: 10.1002/1878-0261.12729 (PMC7400779; doi:10.1002/1878-0261.12729)
Supplement: Supplementary file 8 — Table S1. Primer and TaqMan probe sequences and amplification protocols for cfDNA quantification. Table S2. Total cfDNA concentration in Genome Equivalents pr mL plasma. Table S3. Postoperative change in cfDNA concentration for CRC patients stratified for UICC stage, tumor localization, age and sex. Table S4. Postoperative change in cfDNA concentration in short and long cfDNA fragments. [file MOL2-14-1670-s008.docx]

| Supplementary Table 1 - Primer and TaqMan probe sequences and amplification protocols for cfDNA quantification. All probes contain a 6FAM or HEX fluorophore at the 5’ end and a black hole quencher1 (BHQ-1) at the 3’ end. | | | |
| --- | --- | --- | --- |
| Assay | **Sense primer (5’-3’)**^a^  **Antisense primer (5’-3’)** ^a^  **Probe (5’-3’)** ^a^ | **Amplification Protocol** | **Amplicon size (bp)** |
| Chr3 | CTAGAAGATCTACCTCCAAGAGG  CCAGGCTGAAGCTATTCCAG  CTCATACATCTGGCATATGGGCTGG | 95°C 10’, (95°C 30’‘, 58°C 1‘) x45 & 98°C 10’ | 57 |
| Chr7 | ACATGGGTACTAAGCAACAAAATAAG  CACAATTGGAACATCTTTGTTAAAC  TTGCAGACAAGGTCCCAAAGACAGCA | 95°C 10’, (95°C 30’‘, 58°C 1‘) x45 & 98°C 10’ | 89 |
| CPP-1 | CCATGGATGTATTCGCCAGTTAC  TAAATATTGTGCTTCACCTACTCTAGTG  TTGGCGTAGTTCTCCCGCTTACCCCG | 95°C 10’, (95°C 30’‘, 58°C 1‘) x45 & 98°C 10’ | 94 |
| ^a^ Produced by Sigma Aldrich | | | |

| **Supplementary Table 2 - Total cfDNA concentration in Genome Equivalents pr mL plasma** | | | | | | | |
| --- | --- | --- | --- | --- | --- | --- | --- |
|  | n^a^ | Mean | Min | Q25% | Median | Q75% | Max |
| Colorectal cancer | |  |  |  |  |  |  |
| PreOP | 436 | 4406 | 717 | 2124 | 2958 | 4732 | 48159 |
| Week 1 | 12 | 9326 | 2119 | 5429 | 7369 | 9504 | 25935 |
| Week 2 | 156 | 11878 | 1399 | 4715 | 7754 | 12909 | 116071 |
| Week 3 | 200 | 9832 | 951 | 3592 | 5351 | 10061 | 175981 |
| Week 4 | 48 | 8517 | 1641 | 2539 | 4322 | 7426 | 132663 |
| Week 5 | 13 | 6466 | 1454 | 1975 | 3072 | 7943 | 21682 |
| Week 6 | 7 | 4021 | 2707 | 2896 | 3399 | 4715 | 6818 |
| Bladder cancer^b^ | |  |  |  |  |  |  |
| PreOP | 47 | 2231 | 315 | 1139 | 1662 | 2514 | 10955 |
| Week 1 | 32 | 20962 | 2680 | 7178 | 19961 | 32339 | 64162 |
| Week 2+3 | 7 | 18748 | 4016 | 13917 | 20506 | 24120 | 30643 |
| Week 4 | 23 | 6397 | 822 | 1879 | 4008 | 6615 | 40631 |
| Week 5+6 | 16 | 3431 | 1159 | 1849 | 3160 | 4095 | 10912 |
| ^a^n denotes the number of blood samples taken in the given time period  ^b^Samples in week 2+3 and week 5+6 binned for comparability to Table 2. | | | | | | | |

| **Supplementary Table 3 - Postoperative change in cfDNA concentration for CRC patients stratified for UICC stage, tumor localization, age and sex** | | | | | | | | | | | | |
| --- | --- | --- | --- | --- | --- | --- | --- | --- | --- | --- | --- | --- |
| UICC stage | | | | | | | | | | | | |
|  | I | | |  | II | | |  | III | | | |
| Time | n^a^ | Median ± MAD | P-value^b^ | Time | n^a^ | Median ± MAD | P-value^b^ | Time | n^a^ | Median ± MAD | P-value^b^ | |
| Week 1+2 | 38 | 2.55 ± 1.88 | **<0.0001** | Week 1+2 | 71 | 2.33 ± 1.59 | **<0.0001** | Week 1 | 6 | 3.04 ± 1.72 | **0.0313** | |
|  |  |  |  |  |  |  |  | Week 2 | 53 | 1.94 ± 0.89 | **<0.0001** | |
| Week 3 | 51 | 1.92 ± 1.19 | **<0.0001** | Week 3 | 82 | 1.8 ± 1.07 | **<0.0001** | Week 3 | 67 | 1.71 ± 1.01 | **<0.0001** | |
| Week 4+5+6 | 6 | 1.21 ± 0.54 | 0.3125 | Week 4 | 15 | 1.17 ± 0.44 | 0.1205 | Week 4 | 29 | 1.55 ± 1.07 | **0.0044** | |
|  |  |  |  | Week 5+6 | 8 | 1.15 ± 0.58 | 0.7422 | Week 5+6 | 10 | 1.41 ± 0.94 | 0.1602 | |
|  |  |  |  |  |  |  |  |  |  |  |  |  |
| Tumor localization | | | | | | | | | | | | |
|  | Colon | | |  | Rectum | | |  |  |  |  | |
| Time | n^a^ | Median ± MAD | P-value^b^ | Time | n^a^ | Median ± MAD | P-value^b^ |  |  |  |  | |
| Week 1 | 10 | 3.59 ± 1.16 | **0.002** | Week 1+2 | 43 | 2.44 ± 1.75 | **<0.0001** |  |  |  |  | |
| Week 2 | 115 | 2.03 ± 1.1 | **<0.0001** |  |  |  |  |  |  |  |  | |
| Week 3 | 154 | 1.7 ± 1.01 | **<0.0001** | Week 3 | 46 | 2.11 ± 1.11 | **<0.0001** |  |  |  |  | |
| Week 4 | 37 | 1.24 ± 0.65 | **0.0015** | Week 4+5+6 | 14 | 1.29 ± 0.83 | 0.1353 |  |  |  |  | |
| Week 5 | 11 | 1.16 ± 0.62 | 0.1748 |  |  |  |  |  |  |  |  | |
| Week 6 | 6 | 1.15 ± 0.67 | 0.5625 |  |  |  |  |  |  |  |  | |
| Age | | | | | | | | | | | | |
|  | ≤ Mean | | |  | > Mean | | |  |  |  |  | |
| Time | n^a^ | Median ± MAD | P-value^b^ | Time | n^a^ | Median ± MAD | P-value^b^ |  |  |  |  | |
| Week 1 | 6 | 3.6 ± 1.5 | **0.0313** | Week 1 | 6 | 3.73 ± 1.03 | **0.0313** |  |  |  |  | |
| Week 2 | 68 | 2.35 ± 1.48 | **<0.0001** | Week 2 | 88 | 1.98 ± 1.1 | **<0.0001** |  |  |  |  | |
| Week 3 | 86 | 1.96 ± 1 | **<0.0001** | Week 3 | 114 | 1.68 ± 0.99 | **<0.0001** |  |  |  |  | |
| Week 4 | 23 | 1.23 ± 0.95 | **0.0301** | Week 4 | 25 | 1.24 ± 0.58 | **0.0074** |  |  |  |  | |
| Week 5+6 | 6 | 1.61 ± 0.49 | 0.0938 | Week 5 | 9 | 0.97 ± 0.65 | 0.6523 |  |  |  |  | |
|  |  |  |  | Week 6 | 5 | 0.98 ± 0.5 | 1 |  |  |  |  | |
| Sex | | | | | | | | | | | | |
|  | Male | | |  | Female | | |  |  |  |  | |
| Time | n^a^ | Median ± MAD | P-value^b^ | Time | n^a^ | Median ± MAD | P-value^b^ |  |  |  |  | |
| Week 1 | 7 | 3.34 ± 1.17 | **0.0156** | Week 1 | 5 | 3.86 ± 0.78 | 0.0625 |  |  |  |  | |
| Week 2 | 92 | 2.06 ± 1.28 | **<0.0001** | Week 2 | 64 | 2.18 ± 1.29 | **<0.0001** |  |  |  |  | |
| Week 3 | 111 | 1.85 ± 1.13 | **<0.0001** | Week 3 | 89 | 1.69 ± 0.92 | **<0.0001** |  |  |  |  | |
| Week 4 | 27 | 1.12 ± 0.74 | **0.02** | Week 4 | 21 | 1.24 ± 0.67 | **0.0142** |  |  |  |  | |
| Week 5+6 | 9 | 0.97 ± 0.43 | 0.5703 | Week 5+6 | 11 | 1.63 ± 0.81 | **0.0137** |  |  |  |  | |
| ^a^n denotes the number of blood samples taken in the given time period ^b^Statistically significant P-values (P≤0.05) marked in bold. | | | | | | | | | | | |  |

| **Supplementary table 4. Postoperative change in cfDNA concentration in short and long cfDNA fragments** | | | | | | | | | | |
| --- | --- | --- | --- | --- | --- | --- | --- | --- | --- | --- |
|  |  | **Short fragments** | | | | **Long fragments** | | | |  |
|  | n^a^ | Median ± MAD | Mean | 95% CI | P-value^b^ | Median ± MAD | Mean | 95% CI | P-value |  |
| Week 1 | 2 | 8.15 ± 0.55 | 8.15 | [7.78-8.52] | 0.5 | 1.34 ± 0.29 | 1.34 | [1.14-1.53] | 0.5 |  |
| Week 2 | 30 | 2.76 ± 1.96 | 4.02 | [2.81-5.39] | **<0.0001** | 1.56 ± 1.15 | 2.98 | [1.95-4.17] | **<0.0001** |  |
| Week 3 | 24 | 1.81 ± 1.13 | 2.58 | [1.64-3.71] | **0.0006** | 1.18 ± 0.93 | 2.23 | [1.34-3.43] | 0.0646 |  |
| Week 4 | 23 | 1.41 ± 1.25 | 2.81 | [1.63-4.28] | **0.0094** | 0.86 ± 0.31 | 1.77 | [1.14-2.62] | 0.5009 |  |
| Week 5 | 6 | 1.08 ± 0.74 | 1.91 | [0.68-3.9] | 1 | 1.27 ± 0.62 | 1.27 | [0.89-1.63] | 0.4375 |  |
| Week 6 | 6 | 1.13 ± 0.9 | 3.37 | [0.73-7.08] | 0.4185 | 1.26 ± 0.67 | 2.18 | [0.96-3.66] | 0.2188 |  |
| ^a^n denotes the number of blood samples taken in the given time period ^b^Statistically significant P-values (P≤0.05) marked in bold. | | | | | | | | | | |
